# In vivo dynamics and regulation of DNA G-quadruplex structures in mammals

**DOI:** 10.1186/s13578-023-01074-8

**Published:** 2023-06-28

**Authors:** Ze-Hao Zhang, Sheng Hu Qian, Dengguo Wei, Zhen-Xia Chen

**Affiliations:** 1 Hubei Hongshan Laboratory, College of Life Science and Technology, College of Biomedicine and Health, Interdisciplinary Sciences Institute, Huazhong Agricultural University, Wuhan, 430070 China; 2 College of Science, Huazhong Agricultural University, Wuhan, 430070 China; 3 Shenzhen Institute of Nutrition and Health, Huazhong Agricultural University, Shenzhen, 518000 China; 4 Shenzhen Branch, Guangdong Laboratory for Lingnan Modern Agriculture, Genome Analysis Laboratory of the Ministry of Agriculture, Agricultural Genomics Institute at Shenzhen, Chinese Academy of Agricultural Sciences, Shenzhen, 518000 China

**Keywords:** G-quadruplex, Identification and characterization, Dynamic regulation, Therapeutic applications

## Abstract

G-quadruplex (G4) is a four-stranded helical DNA secondary structure formed by guanine-rich sequence folding, and G4 has been computationally predicted to exist in a wide range of species. Substantial evidence has supported the formation of endogenous G4 (eG4) in living cells and revealed its regulatory dynamics and critical roles in several important biological processes, making eG4 a regulator of gene expression perturbation and a promising therapeutic target in disease biology. Here, we reviewed the methods for prediction of potential G4 sequences (PQS) and detection of eG4s. We also highlighted the factors affecting the dynamics of eG4s and the effects of eG4 dynamics. Finally, we discussed the future applications of eG4 dynamics in disease therapy.

## Introduction

**Fig. 1:**
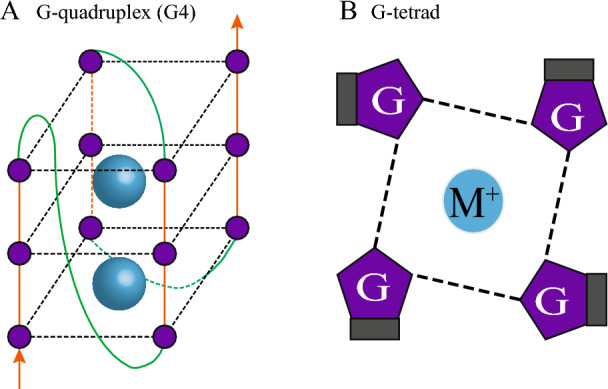
G4 and G-tetrad structures. **A** A typical G4 is formed by assembling four G_3_-tracts (orange) into three G-tetrads, with each G-tetrad stacked on top of another one. The loop sequence (green) connects adjacent G-tracts. Purple circles represent guanosine monophosphate, and blue spheres represent cations. **B** Four guanines, each from one of the four G_3_-tracts, are connected by Hoogsteen hydrogen bonds (black dotted line) to form a square planar plane, the G-tetrad

DNA generally exists as a double helix, and guanine-rich DNA sequences can form four-stranded secondary structures, which are called G-quadruplexes (G4s) (Fig. 1A). A canonical G4 structure is formed by the assembly of two or more G-tetrads, and each G-tetrad consists of four guanines linked by Hoogsteen hydrogen bonds (Fig. 1B) [1]. G4, with its unique secondary structure, is involved in a variety of important biological processes such as gene transcription, translation regulation, telomere extension, and chromatin modification.

The dynamics of endogenous G4s (eG4s), such as folding, unfolding, and their topological changes under different conditions in vivo can affect biological processes, and the detection of eG4s is of great importance for the exploration of their functions. Many methods, including low- or high-throughput methods, have been developed to identify potential G4 sequences (PQS) in silico or in vitro. Fortunately, the advent of cell imaging technology allows the visualization of eG4 folding and unfolding in the context of cell chromatin. With the development of sequencing technology, we can comprehensively identify genome-wide eG4s by ChIP-seq/CUT&Tag methods. The detection methods of eG4s can be used to determine factors affecting eG4 dynamics, including PQS itself and various intracellular factors, and to study eG4 functions.

This review summarizes eG4 detection methods, eG4 characteristics, and self-factors and intracellular factors affecting eG4 dynamics. The effects of eG4 dynamics on telomere extension, transcription, and genomic stability are highlighted. Gene expression can be regulated by eG4 dynamics, suggesting the strong potential of eG4 in biomedical applications.

### Detection and characterization of endogenous G4s

The typical G4 sequence usually consists of four G-tracts of 2–4 bases and three loops connecting adjacent G-tracts. G4 sequences with G-tracts of 2–4 base lengths can form G4 structures with 2–4 G-tetrad layers. Such a sequence feature is used to predict PQS [2, 3] by four algorithms, including regular expression matching algorithms [4, 5], scoring algorithms [6, 7], sliding window algorithms [8, 9], and machine learning algorithms [10]. The algorithms for G4 prediction have been continuously improved, which increases our understanding of eG4s, but the false positive rate of existing algorithms is still high, and so far no software can predict dynamic eG4s, which hinders the study of eG4 function.

Biophysical and chemical methods are used to detect G4 structures in vitro. Classical biophysical methods, including circular dichroism (CD) [11] and nuclear magnetic resonance (NMR) [12], are used to detect the structure of G4s in vitro and to study the dynamics of G4s at different temperatures [13], PEG (polyethylene glycol) concentrations [13], and K^+^ concentrations [14]. The DNA polymerase stop method and the dimethyl sulfate (DMS) and piperidine cleavage method are used to successfully locate the G4s in telomere DNA sequences [15, 16]. Considering that DNA G4s impede polymerase, the high-throughput G4-seq method [17] has been further developed to identify genome-wide DNA G4s in vitro. However, these methods can only be used to detect G4s in vitro, not eG4s in vivo.

Cell imaging provides a strategy to detect and characterize eG4s. The earliest researchers attempted to use G4 antibodies to image intracellular eG4s by immunofluorescence technique. ScFv Sty49 is the first G4-specific antibody that successfully visualized eG4s in ciliate macronuclei [18]. Other antibodies such as scFv hf2 [19], GQ1 zinc finger protein [20], and G4 DARPins [21] are used to detect DNA G4s in vitro, but it turns out that they are unsuitable for whole-cell immunofluorescence assay [21]. There are two reasons for the insensitivity of the above antibodies to eG4s in the cellular chromatin environment. On the one hand, the intracellular chromatin structure covers the immune epitopes of eG4s. On the other hand, the dynamic eG4s can only be folded for a short time under specific cellular conditions [22]. The scFv antibody BG4 [23] generated by phage display and the mouse monoclonal antibody 1H6 [24] generated by immunizing mice with a stable G4 structure have been reported to be used for whole-cell immunofluorescence assay to detect the folding or unfolding of eG4s in vivo. In particular, the cross-reactivity of the G4 antibody 1H6 to immobilized single-stranded T-rich DNA must be considered when using this antibody [25]. Since the generation of the two antibodies depends on the fixed structure of the G4, not all the possible topological structures of eG4s can be detected.

In addition to antibodies, a series of small-molecule fluorescent chemical probes can also be used to detect eG4s. For example (Table 1), probe BMVC [26], squarylium dye TSQ1 [27], and single-molecule fluorescent probe SiR-PyPDS [28] can be used to detect DNA eG4s. ATPD dye [29], NaphthoTASQ [30], and DAOTA-M2 [31] can be used to detect DNA eG4s and RNA eG4s. These probes all have membrane permeability, low cytotoxicity, and high selectivity for eG4s. They can produce strong and specific fluorescence upon binding to eG4s. Small-molecule probes that detect eG4s should not induce the folding of eG4s, but only detect those eG4s that are pre-existing in cells. Currently, TSQ1 and low concentrations of SiR-PyPDS are reported not to induce eG4 folding in vivo [27, 28]. To advance this field, newly developed eG4s probes should ensure that they do not interfere with the dynamics of eG4s and thus detect the actual eG4s rather than spurious eG4s induced by the probe in vivo.

**Table 1:** Examples of G4-specific small-molecule probes

Name	Chemical substance	Detection	Feature and advantage	Refs.
BMVC	3,6-bis (1-methyl-4-vinylpyridinium) carbazole diiodide	DNA eG4s	Can be used to monitor the uptake and localization of guanine-rich oligonucleotides by cells	[26]
TSQ1	Nonlinear optical (NLO) squaraine	DNA eG4s	Cannot induce folding of eG4s. Its excitation and emission lights are harmless to health	[27]
SiR-PyPDS	Silicon-rhodamine (SiR) linked to pyridostatin derivatives (PyPDS)	DNA eG4s	Cannot induce folding of eG4s at low concentrations (20 nM). Capable of single-molecule and real-time detection of individual eG4s in vivo	[28]
ATPD	Anthrathiophenedione	DNA and RNA eG4s	High affinity for DNA and RNA eG4s	[29]
NaphthoTASQ	A fluorogenic naphthalene template surrounded by four synthetic guanine arms	DNA and RNA eG4s	Flexibility in fluorescence wavelength selection due to its red-edge effect (REE)	[30]
DAOTA-M2	Planarized triarylmethyl carbocation (triangulenium) derivatives	DNA and RNA eG4s	Capable of detecting G4s even in the presence of other competing nucleic acid topologies. Suitable for fluorescence lifetime imaging microscope (FLIM)	[31]

Using cell imaging, researchers can observe the dynamics of eG4s in vivo, study the effect of the cellular environment on the dynamics of eG4s, and better link eG4s to their biological functions. However, neither G4 antibodies nor small molecule probes are sufficient to detect all eG4s in vivo. The inability to obtain genome-wide eG4s limits the application of this cell imaging technology.

The combination of cell imaging and next-generation sequencing makes it possible to detect genome-wide eG4s at high resolution. For example, G4 ChIP-seq uses G4 antibodies to pull down eG4-forming regions in vivo [32]. By using fixed cell chromatin as the operation object, the detection results are closer to eG4s in the real chromatin environment, thus expanding the application of the G4 ChIP-seq technique to detect dynamic eG4s in different treatments and different cell lines. For example, when BG4 antibodies were used to isolate eG4-containing fragments from chromatin fragments of human NHEK cells and immortalized HaCaT cells and to construct NGS sequencing libraries, approximately 10,000 DNA eG4s were detected from NHEK cell chromatin, while the number of eG4s detected from immortalized HaCaT cell chromatin was only 1/10 of that from NHEK cells [33]. In addition, BG4-based identification of eG4s and Tn5-based CUT&Tag approaches have been integrated as G4 CUT&Tag [34]. Unlike the G4 ChIP-seq method, which enriches eG4-containing fragments from fixed chromatin fragments, eG4s are bound by BG4 in native cells in the G4 CUT&Tag method. Meanwhile, the Tn5 transposase can generate eG4-containing genomic DNA fragments with sequencing adapters, while most of the extraneous chromatin is not interrupted [35]. Therefore, compared with G4 ChIP-seq, G4 CUT&Tag has simpler operation, higher signal-to-noise ratio, and higher resolution and sensitivity for eG4s detection in vivo. The application of G4 CUT&Tag demonstrates the variability of eG4 dynamics in different cells and the potential association between eG4s and enhancer elements [34, 36, 37]. By mapping eG4s at the genomic level, the distribution of eG4s in cells becomes clearer, and the relationship between eG4 dynamics and eG4 function is further strengthened. The G4 detection methods are listed in Table 2.

**Table 2:** G4 detection methods and their characteristics

Category	Method	Advantage	Limitation	Examples	Refs.
Prediction	Regular expression matching	The potential G4 sequence can be predicted according to the nucleic acid sequences	Dynamic eG4s cannot be predicted by existing prediction tools	Quadparser, Quadruplexes	[4, 5]
	Scoring			QGRS mapper, pqsfinder	[6, 7]
	Sliding window			G4P calculator, G4Hunter	[8, 9]
	Machine learning			Quadron	[10]
Detection and characterization	Biochemical method	The location of G4s in the background of long-chain nucleic acid sequence can be determined	It is impossible to observe G4 dynamics, and most techniques can only detect G4s in vitro	Polymerase stop, DMS and piperidine cleavage	[15, 16]
	Biophysical method	It can characterize the structure of G4 and present G4 dynamics in vitro	Only G4s in short-chain nucleic acid sequences can be detected, and G4s can only be detected in vitro	CD, NMR	[11, 12]
	Cell imaging	The dynamics of eG4s and the intracellular factors affecting eG4 dynamics can be studied in vivo	Not all individual eG4s in the cells can be detected, the resolution needs to be improved, and the detected eG4s lack sequence information	G4 antibody immunofluorescence, G4 small molecular fluorescent probes	[23–31]
Mapping based on NGS sequencing	Polymerase stalling approach	The potential G4 in the genome or transcriptome can be comprehensively detected and the G4 map of the whole genome or transcriptome of the species can be obtained	The operation object is the purified genomic DNA or total mRNA, the environment is artificially controlled, and thus the detection results do not represent the actual eG4s in vivo. It is impossible to study the effect of cellular factors on the G4 dynamics	G4-seq	[17]
	Antibody based pull-down approach	The operation object is fixed chromatin (G4 ChIP-seq) or native cells (G4 CUT&Tag). Genome-wide pre-existing eG4s can be detected in vivo. The effect of cellular factors on eG4 dynamics can be studied	The specificity of antibody binding to eG4s still needs to be improved	G4 ChIP-seq, G4 CUT&Tag	[32, 34]

### Factors affecting eG4 dynamics

During the folding of eG4s, many self-factors will affect the topology. The sequence of eG4 affects its own folding and conformation. The length of the G-tract sequence will affect the conformation of G4s. It has been reported that the human telomere sequence d[TAGG(TTAGGG)_3_] forms hybrid G4s in K^+^ solution, and the silkworm telomere sequence d[TAGG(TTAGG)_3_] with one G missing in each G-tract sequence forms antiparallel G4s in vitro [38]. The loop sequence also affects the topological conformation of G4s. G4s with longer loop sequences tend to form antiparallel structures, whereas G4s with shorter loop sequences tend to form parallel structures [39]. G4 sequences containing two single-base loop sequences will fold into parallel structures [39]. For example, G4 sequences myc-2345, myc-1245, VEGF, HIF-1α, and c-kit21T all have two single-base loop sequences, and NMR detection shows that the above G4 sequences all fold into parallel structures [40, 41], which may be because the short loop sequence is not enough to connect two guanines in the same G-tetrad, but it can connect two guanines in different G-tetrads, as a result, the two G-tract sequences connected by the short loop sequences are parallel.

In addition to the G4 sequence itself (including the G-tract sequence and the loop sequence), flanking sequences and cations are also involved in the folding of eG4s. Cations, including Ca^2+^, Pb^2+^, Sr^2+^, NH^4+^, Na^+^, and K^+^, can coordinate between G-tetrads and participate in the folding of G4s [42–46]. Among these cations, K^+^, which is the most abundant metal cation in mammalian cells [47], has the highest affinity for G-tetrads [48, 49]. The K^+^ environment is favorable for the folding of intermolecular parallel G4s, whereas the Na^+^ environment is favorable for the folding of intramolecular antiparallel G4s. This is because different cations coordinate differently. For example, K^+^ coordinates with guanines in the form of eightfold, while Na^+^ coordinates with guanines in the form of square-planar [39]. The interaction between flanking sequences and loop sequences also affects the folding of eG4 structures. Different flanking sequences interact with loop sequences in different ways, thus forming different base pairs and cap structures and affecting the intra-loop and inter-loop interactions, ultimately affecting the structures of eG4s. For example, under the condition of K^+^-induced folding, the Tel24 sequence can form two conformations: hybrid-1 (dominant) and hybrid-2. When thymine is used to replace A24 (adenine at position 24) on the 3' flanking sequence, Tel24 G4 with hybrid-2 conformation is significantly increased, which is because A24 on the 3' flanking sequence can be paired with T13 base on the loop sequence to form a cap structure, and similarly, T1 on the 5' flanking sequence can be paired with A20 on the loop sequence to form a cap structure. When A24 is replaced by T, the 3' flanking sequence can no longer interact with the loop sequence, so A7 and A8 on the loop sequence are paired to form a cap structure, and similarly, T1 on the 5' flanking sequence is paired with A14 on the loop sequence to form a cap structure, which is conducive to the folding of Tel24 G4 into the hybrid-2 conformation [14].

The structures of eG4s are also affected by interacting proteins in vivo. During DNA replication, dsDNA is unwound into ssDNA by helicases and stabilized by ssDNA-binding proteins. During transcription, the promoter TATA box interacts with TFIIH to melt the promoter. As a kind of nucleic acid structures, eG4s will inevitably be regulated by interacting proteins. The proteins that can interact with eG4s can be divided into two types according to their functions: one is the protein that can unfold eG4s (such as G4 helicase), and the other is the protein that can bind and stabilize eG4s. These two types of proteins together regulate the dynamics of eG4s in vivo.

Most helicases, such as RecQ-like and DEAH box helicase families, can unfold eG4s. Bloom syndrome protein (BLM) and Werner syndrome ATP-dependent helicase (WRN), two of the most important members of the RecQ-like helicase family, can unfold DNA eG4s [50, 51], thereby maintaining genomic stability during DNA replication, repair, and recombination. The mechanism of unfolding eG4s of BLM and WRN is similar, their helicase-RNaseD domain binds to the ssDNA 3' terminal, and the RecQ domain cooperatively binds to eG4s. Finally, eG4s are unfolded in the 3' to 5' direction in an ATP-dependent manner [52]. Both DHX36 and DHX9 of the DEAH-box helicase family have been reported to efficiently unfold eG4s [53–55]. DHX36 unfolds eG4s via the DEAH family helicase mechanism [56]. DHX36 first binds to the 3' end of the nucleic acid chain and unfolds eG4s through a process dependent on ATP shift to the 5' end. In addition, there are Pif1, FANCJ, DNA2, XPD, and ChlR1 helicases, which unfold DNA eG4s from 5' to 3' in an ATP-dependent manner by binding to the 5' tail of DNA [57–61]. In particular, the activity of ChlR1 unfolded antiparallel G4s was much higher than that of unfolded parallel G4s. Most of these enzymes that unfold DNA eG4s are involved in maintaining genomic stability during DNA replication. This reflects the elimination of one of the negative effects of eG4 folding in vivo.

In addition to these helicases, many proteins without helicase activity can also unfold eG4. Cellular nucleic acid-binding protein (CNBP) can bind to eG4s through the zinc finger domain, the CCHC domain, and the RG-rich domain. Recent studies indicate that CNBP can unfold DNA G4s in a variety of proto-oncogene promoters in vitro, and CNBP can regulate gene transcription in vivo by unfolding promoter eG4s [62]. Another transcription factor, MAZ, can promote the unfolding of *HRAS* promoter G4s and their conversion into a B-type double helix, and MAZ is thought to activate *HRAS* transcription by unfolding eG4s [63]. Some proteins can only unfold the eG4s with a specific topology. For example, the ssDNA-binding protein POT1 can only unfold the antiparallel eG4s [64].

Some G4-interacting proteins can induce folding and stabilization of eG4s after binding to eG4. The RBD1 and RBD2 domains of nucleolin have been reported to have a high affinity for the eG4 in the *c-Myc* gene promoter, and thus nucleolin can induce folding and stabilization of eG4, which may be the mechanism by which nucleolin is involved in down-regulating the transcription level of the *c-Myc* gene [65]. LARK can recognize and bind eG4s through the RRM1 and RRM2 domains, thereby inducing folding and stabilization of eG4s, and it can bind many DNA eG4s, such as the eG4s in the promoters of *BmPOUM2*, *c-MYC*, *HIF-1a*, and *c-kit* [66]. The multifunctional DNA repair enzyme APE1 can induce the folding of eG4s and stabilize eG4s, and its loss will not be conducive to the formation of eG4s in vivo [67].

The development of systematic methods for the identification of G4-interacting proteins has promoted the improvement of this field. The genome-wide shRNA screen based on G4 stable ligands contributes to the identification of G4-interacting proteins [68]. This method can systematically identify genes that interact with G4 directly (G4-interacting proteins) or indirectly (proteins involved in G4-dependent pathways). The RNA helicase DDX42 was identified as a G4-interacting protein by the above method and showed a high affinity for RNA G4 in vitro. The co-binding-mediated protein profiling (CMPP) strategy based on the G4 small-molecule photocrosslinking probe can efficiently capture G4-interacting proteins in living cells [69]. SMARCA4 was identified as a G4-interacting protein by CMPP. The high overlap between the genome-wide binding sites of SMARCA4 and DNA eG4s confirmed the results of CMPP. Since SMARCA4 inhibits the formation of the R-loop structure, which is highly co-localized with G4 in vivo [70], SMARCA4 may have the function of unfolding eG4s.

Currently, the regulation of eG4 dynamics by G4-interacting proteins is largely unknown. How G4-interacting proteins regulate the dynamics of specific eG4s at the right time to affect specific biological processes remains poorly understood. We speculated that different proteins might recognize and bind different types of eG4s, or that different G4s might have different mechanisms to recruit specific G4-interacting proteins. However, the above speculation needs to be verified by G4-interacting protein ChIP-seq and other new technologies. Some G4-interacting proteins found in humans are listed in Table 3.

**Table 3:** G4-interacting proteins in humans

Name	Binding	Function	Intracellular role	Refs.
BLM	DNA eG4s	3'–5' unfolding	DNA helicase Involved in DNA replication, DNA repair, and recombination	[52]
WRN	DNA eG4s	3'–5' unfolding	DNA helicase Involved in DNA replication, DNA repair, and recombination	[50]
DHX36	DNA and RNA eG4s	3'–5' unfolding	DNA helicase and RNA helicase Involved in maintaining genome stability and regulating transcription and translation	[55]
DHX9	DNA and RNA eG4s	3'–5' unfolding	DNA helicase and RNA helicase Involved in maintaining genome stability and regulating transcription and translation	[54]
Pif1	DNA eG4s	5'–3' unfolding	DNA helicase Involved in maintaining genome stability and telomere maintenance	[57]
FANCJ	DNA eG4s	5'–3' unfolding	DNA helicase Involved in maintaining genome stability	[58]
DNA2	DNA eG4s	5'–3' unfolding	DNA helicase Involved in the replication of telomeric DNA	[59]
ChlR1	DNA eG4s	5'–3' unfolding	DNA helicase Involved in sister chromatid cohesion, DNA replication, and recombination	[60]
CNBP	DNA and RNA eG4s	Unfolding	CCHC-type zinc finger nucleic acid binding protein, transcription factor Involved in regulation of transcription and translation	[62]
POT1	DNA eG4s	Unfolding	Telomere protection protein Involved in telomere protection and extension	[64]
MAZ	DNA eG4s	Unfolding	Zinc-finger transcription factor Involved in transcription regulation	[63]
XPD	DNA eG4s	Unfolding	Helicase subunit of the general transcription factor TFIIH Involved in transcription initiation and DNA damage repair	[61]
Nucleolin	DNA eG4s	Stablization	Nucleolar protein, transcription factor Involved in transcription regulation	[65]
LARK	DNA and RNA eG4s	Stablization	CCHC-type zinc finger-containing protein, transcription factor Involved in regulation of transcription and translation	[66]
APE1	DNA eG4s	Stablization	Apurinic/apyrimidinic (AP) endonuclease Involved in DNA damage repair and transcription regulation	[67]

**Fig. 2:**
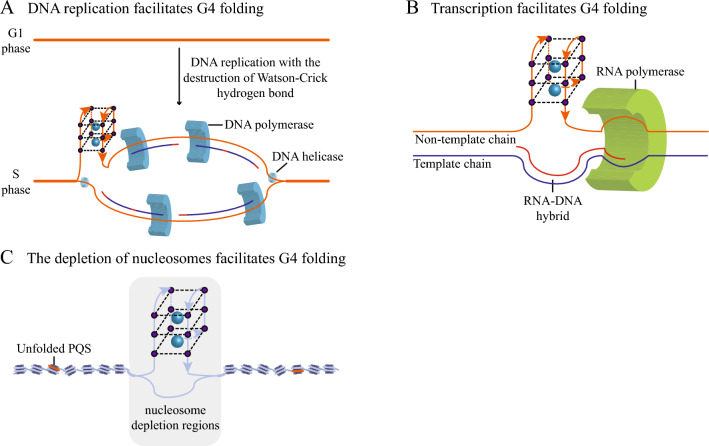
Cellular factors affecting eG4 dynamics. **A** During DNA replication, DNA helicase (blue ring) breaks the Watson–Crick hydrogen bond, resulting in the folding of DNA eG4s in S phase. **B** During transcription, RNA polymerase breaks the Watson–Crick hydrogen bond between the template chain and the non-template chain. When the DNA template chain and RNA form a heterozygous double strand, eG4 folding is induced on the non-template chain. **C** The nucleosome may prevent the Watson–Crick hydrogen bond from being broken, so that PQS cannot fold (orange), and the nucleosome depletion region is more conducive to eG4 folding

In addition to proteins, many cellular factors also influence the dynamics of eG4s in vivo. The folding of eG4s requires the formation of Hoogsteen hydrogen bonds between guanine bases, which makes the folding of genomic DNA eG4s compete with the formation of other nucleic acid structures with Watson–Crick hydrogen bonds. In the processes of DNA replication, transcription, and damage repair, double-strand separation leads to the disruption of Watson–Crick hydrogen bonds, which is conducive to the folding of DNA eG4s. The folding of eG4s is highly likely to occur during DNA replication (Fig. 2A), because the replication mechanism causes the double-stranded DNA to unwind and form a single-stranded DNA, and the single-stranded DNA without the interference of the Watson–Crick hydrogen bond is easier to fold into eG4s [71]. During the cell cycle, it has been reported that eG4 folding reaches its highest level in the S phase with DNA replication [72]. BG4 immunofluorescence shows that in the synchronous cell population, the folding level of eG4 was the lowest in the G0/G1 phase without DNA replication. At the G1/S checkpoint, where DNA replication occurred, the number of eG4s increased 2.5-fold compared to the G0/G1 phase. In the S phase, the maximum number of eG4s was 4.8 times higher than in G0/G1. When aphidicolin was used to inhibit DNA replication, the number of eG4s was reduced by more than twofold, indicating that the folding of eG4s is associated with DNA replication [23]. In a similar study, the fluorescent probe SiR-PyPDS was used to detect the eG4s in the synchronous cell population, and the results showed that during the cell cycle, only 3 eG4s were observed in the G0/G1 phase, 103 eG4s in the G1/S phase, and 208 eG4s in the S phase, indicating that DNA replication in the S phase of the cell cycle promoted the folding of the eG4s in vivo [28]. During gene transcription, heterozygous double-stranded RNA–DNA is formed from newly synthesized RNA and template DNA, which may contribute to the folding of DNA eG4s on non-template chains (Fig. 2B) [73].

Nucleosome depletion may contribute to the folding of eG4s (Fig. 2C). Several studies on the detection and localization of DNA eG4s in chromatin have reported that eG4s are mainly formed in nucleosome-depleted regions and the regulatory regions of transcriptionally active genes [32, 33, 74]. With stem cell differentiation (CNCC, ESC, and NSC) and increased cell specificity, nucleosome-depleted regions disappeared, resulting in the disappearance of eG4s [75].

In addition, telomerase is involved in the unfolding of eG4s in the process of telomere extension. After the expression of telomerase TR subunit and TERT subunit was inhibited by the RNAi method, the accumulation of eG4s was observed in the telomere region, indicating that telomerase or telomerase-associated protein is involved in the unfolding of telomere eG4s in vivo [76].

### Effects of eG4 dynamics

The dynamics of eG4s can regulate gene expression, influence telomere homeostasis, and cause genomic instability.

The dynamics of eG4s can influence gene transcription. Promoter regions are rich in G-rich sequences that form eG4s [77]. The promoter regions of several oncogenes such as *Rb* [78], *RET* [79], *VEGF* [80], *c-Myc* [81], and *bcl-2* [82] could be folded to form G4s in vitro, which was supported by G4 ChIP-seq results of oncogenes such as *PTEN*, *MYC*, *KRAS,* and *TSEN34* [33]. Chromatin DNA eG4s were reported to be enriched at the promoters of highly transcribed genes [33, 34]. Transcriptional downregulation of *MYC*, *KRAS*, and *KIT* genes was observed after cells were treated with G4 stable ligands [83–85]. These results further support the relationship between transcription and eG4s.

**Fig. 3:**
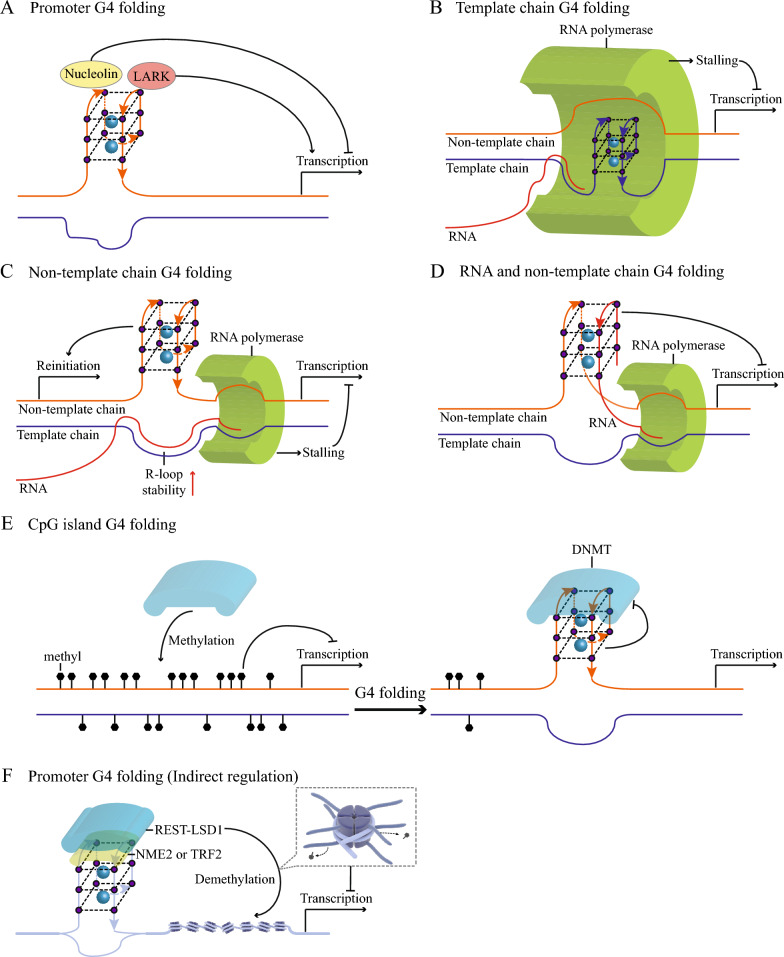
Effects of eG4 dynamics on transcription. **A** The promoter binds to various transcription factors through eG4 folding to regulate gene transcription. **B** Folding of template chain eG4s leads to the stalling of RNA polymerase and reduces the level of transcription. **C** Folding of non-template chain eG4s has two effects on transcription. Folding of eG4s improves the structural stability of the R-loop formed by template chain DNA and RNA, thereby reducing transcription levels. In addition, folding of eG4s also maintains the separation of template chain and non-template chain, thereby promoting transcription reinitiation. **D** Folding of heterozygous eG4s, consisting of RNA and non-template chain DNA, leads to advanced transcription termination and decreased transcription levels. **E** DNMT methylates DNA using CpG as a target, and transcription of the methylated DNA is inhibited (left). When eG4s are folded on the CpG island, they bind DNMT and inhibit its activity, thereby reducing the level of methylation and avoiding transcription inhibition (right). **F** Promoter eG4 folding also indirectly regulates transcription levels. The REST-LSD1 complex is recruited to eG4s in an NME2- or TRF2-dependent manner. Histones of nearby nucleosomes are demethylated by REST-LSD1, resulting in transcription repression

The eG4 dynamics can regulate gene transcription by altering the binding between the transcription factors and the promoters (Fig. 3A). Early analyses based on computational methods have shown that transcription factor-binding motifs are enriched in some promoter G4 motifs [86]. The transcription factor nucleolin can bind and stabilize G4 in the *MYC* promoter in vitro [65]. After their folding, eG4s recruit the transcription factor nucleolin to bind to the *c-MYC* promoter, thereby inhibiting gene expression [87]. NM23-H2 can bind and unfold the *MYC* promoter eG4s [88]. Based on these findings, it has been speculated that nucleolin and NM23-H2 may be involved in stabilizing and unfolding *MYC* promoter eG4s, respectively, to regulate *MYC* transcription. A number of transcription factors, including CNBP [89], SP1 [90], and LARK [66], interact with G4 in vitro. A recent study showed that after their folding, the eG4s in the *BmPOUM2* gene promoter recruit the transcription factor LARK to activate the transcription of the *BmPOUM2* gene in the silkworm [66]. The role of some eG4s in the coding region of the gene is similar to that of promoter eG4s. For example, the folding of eG4s in the first exon of the human *hTERT* gene prevents the binding of the CCCTC-binding factor to the *hTERT* gene, resulting in increased transcription of the *hTERT* gene [91].

The eG4s in genes regulate gene transcription in several ways. The folding of DNA eG4s on the gene template chain tends to inhibit the transcriptional elongation of the genes (Fig. 3B). For example, in human embryonic kidney cells and *Escherichia coli*, the folding of eG4s on the template chain hinders the elongation of RNA polymerase, thereby reducing the transcription level of the corresponding gene [92, 93]. Folding of DNA eG4s on the non-template chain can regulate the transcription level of the gene (Fig. 3C). On the one hand, the non-template chain eG4s hinder the renaturation between the template chain and the non-template chain, which helps to improve the stability of the R-loop structure formed by the template chain and the RNA. The stable R-loop structure causes the polymerase to stall, preventing normal RNA polymerase-mediated transcription. Insertion of the G-rich sequence into the non-template chain resulted in a decrease in T7 polymerase transcription levels, suggesting that eG4s may contribute to R-loop-mediated transcription inhibition [91]. On the other hand, the non-template chain eG4s may be beneficial for transcription restart because they can keep the template chain and non-template chain unwound. Human genes containing more G4 sequences on the non-template chain within 500 bp downstream of the transcription start sites showed higher transcription levels and RNA PolII occupancy rates [94]. Folding of intermolecular eG4s derived from the non-template chain and RNA leads to premature termination of transcription, thereby reducing the level of gene transcription (Fig. 3D). RNA and non-template DNA chains generated from the transcription of the mitochondrial gene *CSBII* fold into stable DNA-RNA heterozygous eG4s to promote transcription termination [95]. Insertion of the DNA-RNA heterozygous eG4-forming sequence into the reporter plasmid resulted in the inhibition of plasmid transcription [96].

In addition to the direct regulation methods mentioned above, eG4s can also regulate gene transcription through epigenetic modifications. Chromatin is mainly composed of DNA and histones. On the one hand, the dynamics of eG4s regulate DNA methylation (Fig. 3E). DNA methyltransferase catalyzes the formation of 5-methylcytosine at CpG dinucleotide sites. DNA methyltransferase prefers to bind DNA G4 over double-helix DNA in vitro [97]. When folded, DNA eG4s recruit DNA methyltransferase 1 (DNMT1) and inhibit its activity, which is confirmed by the high hypomethylation of the DNMT1 binding site of eG4s in human leukemic cells [98]. DNA methylation reduces the accessibility of DNA and thus inhibits gene expression, while eG4 folding avoids DNA methylation-induced transcriptional downregulation through the above mechanism. On the other hand, the dynamics of eG4s are involved in the regulation of nucleosome histone modification (Fig. 3F). After folding, eG4s in the *hTERT* gene promoter recruit the REST-LSD1 (RE1-silencing transcription factor and lysine-specific histone demethylase 1A) complex by binding to NME2, so that the histone H3 Lys4 near the promoter is demethylated, which makes the chromatin structure compact and eventually inhibits gene transcription [99]. The *CDKN1A* gene has a similar mechanism. After folding, eG4s in the *CDKN1A* promoter bind TRF2 to recruit the REST-LSD1 complex, resulting in histone demethylation, which ultimately leads to transcriptional downregulation [100].

**Fig. 4:**
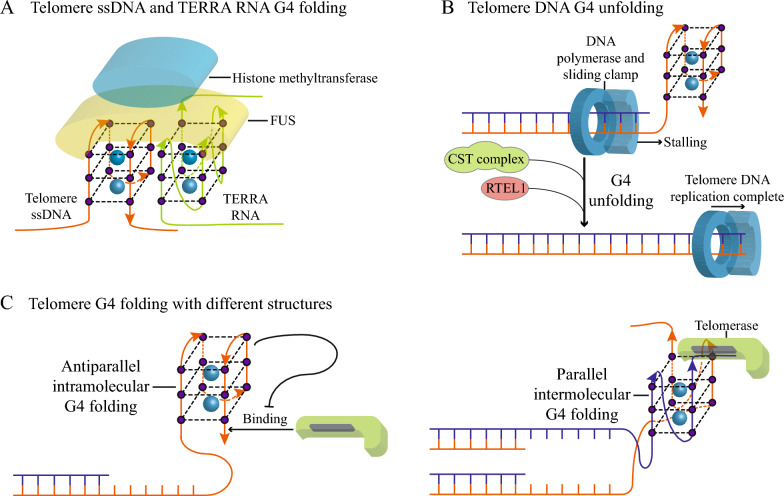
Effects of eG4 dynamics on telomeres. **A** Folded telomere eG4s (orange) and TERRA RNA eG4s (green) together serve as a scaffold to recruit FUS protein and histone methyltransferase to maintain the state of telomere heterochromatin. **B** Normal telomere DNA replication requires telomere eG4s to be unfolded by the CST complex or RTEL1. **C** Telomere eG4s are folded into different structures and regulate telomere extension. Antiparallel intramolecular eG4s inhibit telomerase binding and telomere extension (left). Parallel intermolecular telomere eG4s promote telomerase-mediated telomere extension (right)

Endogenous G4s are also found in telomeres [23, 101] located at the end of chromosomes, and the maintenance of telomere homeostasis depends on the folding of eG4s (Fig. 4A). After binding to eG4s in telomeres, the long non-coding RNA TERRA can be used as a platform for telomere-protein binding [102]. In addition, FUS protein binds to TERRA eG4s and telomere eG4s to recruit histone methyltransferases that maintain the state of telomere heterochromatin. The methyltransferase methylates the histone of telomere nucleosomes to compact the structure of telomere chromatin and maintain the state of heterochromatin. Meanwhile, telomere eG4s prevent DNA polymerases from replicating (Fig. 4B). Many proteins, such as the CTC1-STN1-TEN1 (CST) complex [103] and regulator of telomere elongation helicase 1 (RTEL1) [104], can unfold telomere eG4s, thereby preventing telomere eG4-induced reduction in telomere replication rate, telomere shortening, and abnormal telomere formation [105]. When the CST complex is depleted and eG4s is stabilized by PDS, telomeres are lost in vivo [103]. In addition, different topologies formed by eG4 folding can regulate telomere extension by telomerase (Fig. 4C), a reverse transcriptase composed of RNA and protein. In cancer cells and stem cells with high division and proliferation capacities, telomerase uses its own RNA component as a template to reverse transcribe and extend the 3' end of chromosomal telomere DNA to prevent DNA replication-induced telomere shortening. Folding of the antiparallel intramolecular telomere DNA eG4s restricts the binding of the telomere 3' end to telomerase and inhibits telomere extension [106]. The parallel intermolecular telomere DNA eG4s formed in the S phase of the cell cycle are the substrate and localization site of telomerase, which contributes to telomere extension [107].

In addition, the folding of DNA eG4s increases the likelihood of DNA mutations, leading to genomic instability. *Caenorhabditis elegans* strains with deletions in the *DOG-1* gene (whose product is a homolog of the FANCJ helicase and can unfold DNA eG4s) accumulate gene mutations with base deletions in the G-rich regions that form eG4s [108]. This may be because the folded eG4s interfere with the progression of DNA replication forks, thus causing replication stalling and DNA double-strand breaks, which may lead to gene mutations in the process of DNA double-strand break repair [109, 110], which is supported by the following studies. A previous study found that deletion of the FANCJ helicase or the presence of G4 stable ligands leads to replication stalling at eG4s [111]. A recently developed DSB detection technique called i-BLESS has detected DNA double-strand breaks at eG4s [112]. Genetic analysis of *Saccharomyces cerevisiae* shows that the G4 helicase Pif1 can unfold eG4s and inhibit DNA damage and chromosome recombination caused by eG4s [113]. The FANCJ helicase and the ssDNA-binding protein RPA can jointly promote the unfolding of DNA eG4s to ensure DNA replication in the S phase of the cell cycle [58]. BLM, WRN, and FANCJ jointly mediate DNA eG4 unfolding, thereby reducing the negative effects of eG4 folding in vivo and maintaining genome stability to some extent [114].

### Regulation of eG4 dynamics in therapeutic applications

Because eG4 dynamics are involved in many biological processes in cells as described above, they affect some obvious characteristics of organisms. As mentioned above, eG4s are enriched in proto-oncogene promoters and telomeres, and thus eG4s are associated with cancer.

**Fig. 5:**
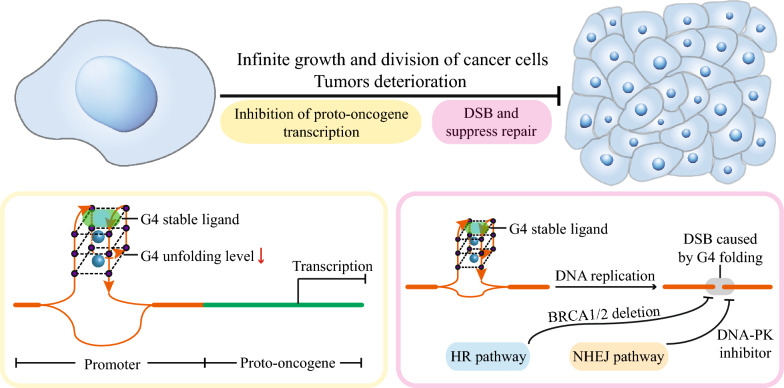
Regulation of eG4 dynamics and cancer treatment. Cancer cells grow and divide indefinitely in vivo, leading to tumor growth and disease progression. There are two potential ways to target G4 in cancer treatment. The first method is to use G4-stabilizing ligands to prevent the unfolding of the proto-oncogene promoter eG4s, thereby reducing the expression of proto-oncogenes in cancer cells (yellow). The second method is to kill cancer cells by combining DSBs caused by G4-stabilizing ligands with inhibition of the HR or NHEJ pathway (magenta)

First, the overexpression of proto-oncogenes will lead to abnormal cell proliferation and cause the transformation of normal cells into cancer cells. The G4-stabilizing ligands or eG4 stabilizers can inhibit the upregulation of proto-oncogenes at their promoters, and they have great potential for cancer treatment (Fig. 5)*.* A previous study has shown that the tri-substituted naphthalene diimide G4-targeting ligand CM03 exhibits anticancer activity on the pancreatic ductal adenocarcinoma cell line and the patient-derived xenograft (PDX) model. RNA-seq analysis shows that CM03 down-regulates many PQS-rich genes associated with cell proliferation, metastasis, and drug resistance in cancer cells [115]. In addition, the tetra-substituted naphthalene diimide derivative MM41 has been reported to exhibit anticancer activity against the MIA PaCa-2 pancreatic cancer PDX model and to reduce tumor growth by 80% in tumor-bearing mice [116]. This may be due to the fact that MM41 can bind and stabilize the promoter eG4s of proto-oncogenes such as *BCL-2* and *k-RAS*, resulting in approximately 40% and 30% downregulation of their transcription, respectively. The G4-stabilizing ligand PDS has also been reported to inhibit the transcriptional expression of several oncogenes [117].

Second, the expression of telomerase is activated in cancer cells, thus resulting in the repair of telomere DNA shortening caused by the continuous division of cancer cells. The G4 stabilizer can inhibit the unfolding of telomere eG4s, thereby inhibiting telomerase-mediated telomere DNA extension in cancer cells and ultimately leading to cancer cell death [118].

Finally, DNA double-strand breaks (DSBs) caused by eG4 dynamics have also been used to treat cancer (Fig. 5). The folding of eG4s in the genome leads to abnormal DNA replication, resulting in DSBs. Normally, DSBs are repaired by homologous recombination (HR) and non-homologous end joining (NHEJ). When DSBs are induced by the G4 stabilizer in cancer cells and cannot be repaired by the HR or NHEJ pathways, the cancer cells are effectively killed. For example, the G4 stabilizer CX-5461 can induce eG4 folding and stabilize eG4s in BRCA1/2-deficient cancer cells, thereby inducing replication-dependent DSBs and ultimately killing cancer cells [119]. BRCA1/2 is an important DNA damage repair protein involved in the HR pathway, and after DSBs, BRCA1/2-deficient cancer cells die because DSBs cannot be repaired by the HR pathway. The G4 stabilizer CX-5461 has been reported to have good anticancer effects, and it can inhibit the growth of the BRAC2-deficient colon cancer cell line HCT-116 and suppress triple-negative breast cancer in a PDX model with mutations in the *BRAC1* and *BRAC2* genes. Inhibition of the NHEJ pathway can also play a similar role. DNA-PK is an important protein in the NHEJ pathway and is involved in the repair of broken DNA ends. The anticancer effect of the combination of the G4-stabilizing ligand PDS and the DNA-PK inhibitor NU7441 on the human fibrosarcoma cell line HT1080 is increased by about 45% compared to the use of PDS alone [120].

## Conclusion

As critical nucleic acid structures, eG4s endow DNA with some additional functions, such as participation in transcription regulation, DNA methylation, and histone modification. The dynamics of eG4s have two opposing effects on the intracellular chromatin DNA. On the one hand, the folding of eG4s is very important for telomere structure to maintain the independence and integrity of linear DNA. On the other hand, the unfolding of eG4s is a factor that contributes to the stability of genomic DNA. Since eG4 dynamics have a variety of biological functions in cells, the use of G4-stabilizing ligands or G4 stabilizers to regulate eG4 dynamics has been confirmed as an effective therapy for cancer. In recent years, eG4 research methods have continuously evolved from various extracellular biophysical methods to the application of cell imaging and high-throughput sequencing for genome-wide detection of eG4s. The above methods have been used to investigate the factors affecting the dynamics of eG4s, the critical roles of eG4 dynamics in cells, and the interacting proteins of eG4s. However, there is still much room for improvement in the resolution and sensitivity of existing methods for studying eG4 dynamics. In the future, as research technology continues to improve, the influence and molecular mechanism of eG4 dynamics in cells will be further explored.

## Data Availability

Not applicable.
